# "London" Nurses and Their Emoluments

**Published:** 1918-01-26

**Authors:** Eva C. E. Luckes

**Affiliations:** Matron. London Hospital, Whitechapel, E.


### THE EDITOR'S LETTER-BOX.

### "London" Nurses and their Emoluments.

*To the Editor of* THE HOSPITAL.

SIR,—I think it is only right to draw your attention to an error occurring in this week's edition of THE HOSPITAL, January 19, on page 338, paragraph 3, in which it is stated "that the London Hospital private staff nurses, who were formerly paid from £24 to £27 per annum, have all commenced their fourth year with a salary of £30, rising to £50 by increases of £5 a year," etc.

Private staff nurses always had a salary commencing at £30 per annum. A nurse having been appointed on the private nursing staff at the end of her two years' training has formerly started with a salary of £30 the first year of service, £35 the second year, rising to a maximum of £45. They now start at £35 per annum, rising by £5 annually to a maximum of £50, instead of £45 as previously.

In addition, every member of the nursing staff receives £5 per annum to her salary after six years from the date of her entrance as a probationer—*i.e.* this makes a private nurse's salary £55 per annum. After twelve years' service a second increase of £5 per annum is given. They also receive an additional £5 annually to their salary if they have paid for their midwifery training at the London and hold the C.M.B. certificate, whether they undertake midwifery duties or not.—Faithfully yours, EVA C. E. LUCKES, Matron.

London Hospital, Whitechapel, E.,

January 19, 1918.

[We thank Miss Luckes for the correction. The figures were correctly given on page 210 of THE HOSPITAL of December 9, 1916, and we regret that in the summary which Miss Luckes corrects the staff nurses and private staff nurses got mingled.—ED. THE HOSPITAL.]

